# Genetic line-specific immune profiles and immunometabolic responses to intramuscular lipopolysaccharide injection

**DOI:** 10.3389/fimmu.2025.1608391

**Published:** 2025-06-16

**Authors:** Kayla M. Elmore, Susan J. Lamont, Elizabeth A. Bobeck

**Affiliations:** Department of Animal Science, Iowa State University, Ames, IA, United States

**Keywords:** cellular metabolic profile, genetic line, immune cell profile, immunometabolic assay, lipopolysaccharide, poultry

## Abstract

Previous research has investigated highly inbred chicken genetic lines from a metabolic, immune response, genetic profile, and immune trait standpoint, including response to lipopolysaccharide (LPS). Fayoumi lines (M5.1, M15.2) are known for their resistance to bacterial and viral infections, while Leghorn lines (Ghs6, Ghs13) display lower disease resistance. Results highlighted a need to increase LPS dose above initial work using 1mg/kg bodyweight (BW). Therefore, this study investigated the immune profiles and metabolic phenotypes of peripheral blood mononuclear cells (PBMC) from highly inbred genetic lines under resting and stressed metabolic states. Fifty-four adult birds from 5 highly inbred genetic lines (M5.1, M15.2, Ghs13, Line-8, and Sp-21.1) were randomly assigned to 0.9% sterile saline control or 2.4 mg/kg BW intramuscular LPS (*Escherichia coli* O55:B5). BW was recorded at baseline before injection and 24 h post-injection (hpi). Cloacal temperature was recorded at baseline, 2 hpi, and 24 hpi, while blood was collected for flow cytometry and metabolic analysis. Data were analyzed using the SAS 9.4 MIXED procedure with genetic line, injection status, and interaction as fixed effects, with significance at *p* ≤ 0.05. Baseline immune cell profiles varied by line (*p* ≤ 0.001). At 2 hpi, LPS did not impact BW or temperature, but influenced all queried immune cell populations while decreasing ATP production and glycolytic rates (*p* ≤ 0.02). At 2 hpi, M5.1, Line-8, and Sp-21.1 LPS-inoculated birds had increased circulating CD3^+^ cells (51.8-62.3%, *p* ≤ 0.0001). LPS decreased CD3^+^CD1.1^+^ cell levels by 34.1% at 2 hpi (*p* ≤ 0.0001). M5.1, M15.2, and Line-8 controls had 14.9-66.5% higher CD3^+^CD4^+^ levels than LPS-inoculated birds, while CD3^+^CD4^+^ cells were 12.2% lower in Ghs13 post-LPS (*p* ≤ 0.0001). CD3^+^CD8α^+^ populations increased 41.1-63.2% in all LPS-injected birds at 2 hpi, except Ghs13 (*p* ≤ 0.0001). These results highlight genetic line-specific immune responses to LPS. By 24 hpi, immune profiles and glycolytic rates were largely recovered from LPS, while genetic line effects persisted, indicating line-specific immune responses (*p* ≤ 0.04). Further understanding cellular preference and metabolic switching during inflammatory challenges could provide insight into how to best support and optimize bird performance during the production cycles.

## 1 Introduction

Extensive research on genetic profiling and immune traits has been conducted using highly inbred genetic chicken lines, including the Fayoumi lines M5.1 and M15.2 and Leghorn lines Ghs6 and Ghs13 (1). These genetic lines have contributed to key discoveries in several areas, including but not limited to heat tolerance, immune markers, immune function, disease resistance, antibody production, and antibody genetic markers (1–4). Evaluating gene expression has allowed researchers to describe the Fayoumi lines as being relatively resistant to viral diseases, such as Avian Influenza Virus (AIV) and Newcastle Disease Virus (NDV), compared to Leghorn lines (3, 5). This resistance is thought to be a result of upregulated hemoglobin family genes, thus increasing oxygen carriers to help maintain normal blood chemistry (5). Additionally, mRNA expression in bone marrow-derived dendritic cells from Fayoumi lines (M5.1) showed a reduced heat-induced inflammatory response compared to Ghs6, suggesting Fayoumi lines may be able to maintain homeothermy more adequately than Leghorn lines (4, 6, 7). Bone marrow derived dendritic cells from the M5.1 and M15.2 lines have also been shown to be more resistant to lipopolysaccharide (LPS) than the Ghs6 line due to increased nitric oxide production, higher MHC II surface expression, and increased phagocytic capability (4, 6). When evaluating differences within genetic line, Fayoumi lines were relatively resistant to avian coccidiosis, with M5.1 being more resistant than M15.2 (*Eimeria maxima (* 8 *)*; *Eimeria tenella (* 9 *)*). Within the Leghorn lines, Ghs13 is known to be more resistant to NDV than Ghs6, while the two lines have not been investigated using a bacterial infection model (5, 10). Therefore, Ghs13 was expected to perform similarly to Ghs6 (7, 10).

While Leghorn Line-8 and Spanish-21.1 (Sp-21.1) inbred lines have not been as thoroughly investigated as Fayoumi and Ghs Leghorn lines, acute LPS exposure has been previously investigated in the Ghs, Line-8, and Sp-21.1 genetic lines at an intramuscular dose of 1 mg/kg BW (11). LPS is an outer component of gram-negative bacteria, such as *Escherichia coli*, and is widely used in research to model acute and chronic inflammatory responses (12). Using a similar study design, previous results suggested that genetic line may have a greater influence on metabolic pathway preferences than LPS injection at 6 and 24 hours post-injection (hpi) (11). While fever responses were not found due to LPS, the Ghs line displayed delayed immune cell recruitment following LPS injection but resolved LPS-induced inflammation by 24 hpi. Additionally, Line-8 had a strong baseline T-cell presence and balanced immune and metabolic response following LPS challenge. Sp-21.1 line had a robust T cell response and glycolytic metabolic activity, which may benefit rapid, energy-intensive immune responses (11). All genetic lines showed metabolic recovery by 24 hpi despite evident genetic line differences (11). The previous experimental designs concluded that genetic background influenced the scale and efficiency of immune activation and resolution. A future goal, therefore, was to investigate increased dosages and query earlier timepoints to determine if line-specific immune preferences and responses may be more pronounced at earlier activation timepoints post-LPS exposure, further providing insight into genetic background influence on bacterial resistance.

For example, 18-wk-old New Hampshire and Rhode Island Red pullets experienced severe hypothermia within the first 4 h, while a White Leghorn line had severe hyperthermia as early as 4 h following a 2 mg/kg LPS intramuscular injection (13). In 12-wk-old Rhode Island Red and New Hampshire cockerels, a 2 mg/kg intramuscular LPS dose had been shown to significantly decrease rectal body temperature at 3 hpi, leading to severe hypothermia at 5 hpi, and temperatures resolved at 24 hpi (14). In contrast, 12-wk-old White Leghorn cockerels elicited severe biphasic hyperthermic responses at 2 hpi, lasting 2–4 h following an identical LPS dose (14). Therefore, a 2 hpi sampling timepoint was selected in the present study to capture LPS-induced body temperature changes across varying genotypes.

Based on previous work from this lab investigating PBMC immune profiles and cellular metabolism of highly inbred and advanced intercross genetic lines following an intramuscular LPS injection at a 1 mg/kg dose (*Escherichia coli* O55:B5 (11)), the authors chose to continue to use a non-infectious LPS model with an intramuscular dosage of 2.4 mg/kg BW to further stimulate a physiological response and enhance immune profiles and immunometabolic phenotypes. Therefore, the objectives of this study were to assess immune profiles and metabolic phenotypes of peripheral blood mononuclear cells (PBMC) in 5 highly inbred genetic lines (M5.1, M15.2, Ghs13, Line-8, and Sp-21.1) of adult chickens before and after a single 2.4 mg/kg BW intramuscular LPS injection.

## 2 Materials and methods

### 2.1 Animals and LPS administration

All experimental procedures were approved by the Iowa State University Institutional Animal Care and Use Committee #22-113. This study utilized 54 adult birds representing five distinct highly inbred genetic lines (M5.1, M15.2, Ghs13, Line-8, and Sp-21.1) sourced from an existing breeding colony at Iowa State University’s Robert T. Hamilton Poultry Teaching and Research Facility (Ames, IA). The birds were ~127–130 weeks old. Not all genetic lines could be replicated from previous work due to bird availability (11, 15). All birds were individually housed in hanging cages with continuous access to a standard laying hen diet and water. On day 0, birds from each genetic line (n = 10 per line) were randomly assigned to one of two injection treatments, creating a 5 × 2 factorial design of genetic line (M5.1, M15.2, Ghs13, Line-8, and Sp-21.1) by injection. Each bird received an intramuscular injection administered in equal portions across the left and right breast and thigh muscles. Treatments consisted of either 2.4 mg/kg BW lipopolysaccharide (LPS, *Escherichia coli* O55:B5; Sigma-Aldrich, St. Louis, MO) or an equivalent volume of 0.9% sterile saline. Body weight was recorded at baseline before injection and 24 h post-injection (hpi). Cloacal temperature measurements were recorded at baseline, 2 hpi, and 24 hpi.

### 2.2 Sample collection

At each timepoint, 1 ml of blood was collected from the brachial vein of all birds using heparinized syringes and collection tubes. Blood was diluted to a 1:1 ratio with sterile phosphate-buffered saline (PBS), and PBMC were isolated following previously published methods (15, 16). Briefly, PBMC were isolated using layered Histopaque 1077 and 1119 density gradient (Sigma Aldrich, St. Louis, MO), centrifugation, and washed with sterile PBS. For Seahorse metabolic assays, PBMC were resuspended in Seahorse XF DMEM medium (pH 7.4, 37°C; Agilent, Santa Clara, CA) and counted by hemocytometer. Fresh PBMC was used in Seahorse XF metabolic assays. A mixture of heat-inactivated chicken serum (Equitech-Bio Inc., Kerrville, TX) and DMSO (Thermo Fisher Scientific, Waltham, MA) was added to the remaining PBMC and stored at -80°C for flow cytometry.

### 2.3 Cytometric and metabolic assays

Flow cytometric and metabolic assays were performed following previously published methods (15, 16). Briefly, PBMC from all timepoints were thawed and washed in RPMI-1640 medium (Cytiva-Hyclone, Logan, UT), resuspended in PBS (Corning, Corning, NY), and aliquoted evenly across 6 flow cytometry tubes (Corning, Corning, NY). All samples were stained using fluorescence-minus-one controls and corresponding isotype controls. The panel used in the current study included: PE anti-chicken monocyte/macrophage (clone KUL01; mouse IgG1κ), Pacific Blue™ anti-chicken CD3 (clone CT-3; mouse IgG1κ), FITC anti-chicken CD1.1 (clone CB3; mouse IgG1κ), PE/CY7 anti-chicken CD4 (clone CT-4; mouse IgG1κ), Alexa Fluor^®^ 700 anti-chicken CD8α (clone CT-8; mouse IgG1κ; Southern Biotech, Birmingham, AL; (15)). All samples were stained in the dark for 30 min at 4°C. Samples were analyzed using a BD FACSCanto™ cytometer (BD Biosciences, San Jose, CA), and gating strategies were generated using FlowJo version 10.5.0 software (BD Biosciences, San Jose, CA).

For Seahorse XF metabolic assays, fresh PBMC isolated from each baseline sample were plated in quadruplicate, and all PBMC from 2 hpi and 24 hpi were plated in triplicate at 200,000 cells/well for use in the Seahorse XF Real-Time ATP Rate Assay and Glycolytic Rate Assay kits on the Seahorse XFe96 Analyzer (Agilent, Santa Clara, CA). Agilent User Guides and protocols were followed to perform metabolic tests.

### 2.4 Statistical analysis

Outliers in each dataset were identified using the UNIVARIATE procedure. Data were analyzed separately at each timepoint using a mixed linear model and Tukey-Kramer adjustment to correct for multiple comparisons (PROC MIXED, SAS 9.4, Cary, NC). The model included fixed effects for genetic line, injection status, and their interaction across all timepoints (baseline, 2 hpi, and 24 hpi). For flow cytometric data, tube effects were treated as a random factor. Least-square means and standard errors (SEM) were calculated for all variables and statistical significance was determined at *p* ≤ 0.05.

## 3 Results

### 3.1 Body weight and temperature

LPS injection had no significant impact on bird body weight or temperature at any timepoint (*p* ≥ 0.05, **Table 1**). The observed differences in body weight among genetic lines are an inherent trait of their genetic background.

**Table 1:** Body weight and temperature of 5 highly inbred genetic lines of adult birds (M5.1, M15.2, Ghs13, Line-8, and Sp-21.1) administered ± 2.4 mg/kg lipopolysaccharide injection at baseline, 2 hpi, and 24 hpi.

Measure	M5.1		M15.2		Ghs13		Line-8		Sp-21.1		Adj. *p*-value		
	CON	LPS	CON	LPS	CON	LPS	CON	LPS	CON	LPS	Line^1^	Trt^2^	Line^1^xTrt^2^ Trt^2^Trt^2^
Body weight (kg)													
Baseline	1.33^b^	–	1.66^a^	–	1.47^ab^	–	1.49^ab^	–	1.65^a^	–	0.03	–	–
Pooled SEM	0.08	–	0.08	–	0.07	–	0.07	–	0.08	–	–	–	–
24 hpi	1.18	1.34	1.97	1.63	1.53	1.39	1.43	1.57	1.57	1.73	0.20	0.91	0.78
Pooled SEM	0.22	0.22	0.18	0.18	0.18	0.18	0.18	0.18	0.22	0.22	–	–	–
Δ24 hpi	-0.04	-0.04	-0.03	-0.04	-0.02	-0.06	-0.04	-0.04	-0.02	-0.06	–	–	–
Average SEM	0.004	0.02	0.001	0.01	0.01	0.04	0.02	0.01	0.003	0.06	–	–	–
Temperature (C°)													
Baseline	40.99	–	40.78	–	41.00	–	40.93	–	40.84	–	0.61	–	–
Pooled SEM	0.12	–	0.12	–	0.11	–	0.11	–	0.12	–	–	–	–
2 hpi	40.96	41.39	40.89	40.63	41.00	40.67	40.85	40.63	40.74	40.85	0.63	0.81	0.73
Pooled SEM	0.30	0.30	0.37	0.37	0.30	0.30	0.30	0.30	0.30	0.30	–	–	–
24 hpi	41.00	41.80	41.05	41.10	41.17	41.13	41.13	40.70	40.80	40.55	0.32	0.86	0.44
Pooled SEM	0.31	0.31	0.26	0.26	0.26	0.26	0.26	0.26	0.31	0.31	–	–	–
Δ6 hpi	-0.17	0.48	-0.11	0.17	-0.06	-0.35	-0.19	-0.41	0.02	0.06	–	–	–
Average SEM	0.20	0.41	0.50	0.17	0.23	0.58	0.20	0.60	0.10	0.20	–	–	–
Δ24 hpi	-0.14	0.89	0.33	0.19	0.22	0.15	0.06	0.26	0.08	-0.61	–	–	–
Average SEM	0.42	0.17	0.17	0.11	0.00	0.48	0.34	0.23	0.25	0.06	–	–	–

Data represent the mean and SEM for all genetic lines at each timepoint (n = 12 birds for Ghs13 and Line-8 lines, and n = 10 birds for M5.1, M15.2, and Sp-21.1 lines at baseline; n = 3 birds/treatment for M5.1, Ghs13, Line-8, and Sp-21.1 lines, and n = 2 birds/treatment for M15.2 line at 2 hpi; n = 3 birds/treatment for M15.2, Ghs13, and Line-8 lines, and n = 2 birds/treatment for M5.1 and Sp-21.1 lines at 24 hpi). Different letter superscripts within a timepoint indicate significance at *p* ≤ 0.05.

^1^Line, Genetic line main effect.

^2^Trt, Challenge main effect.

### 3.2 Flow cytometry

**Figure 1:**
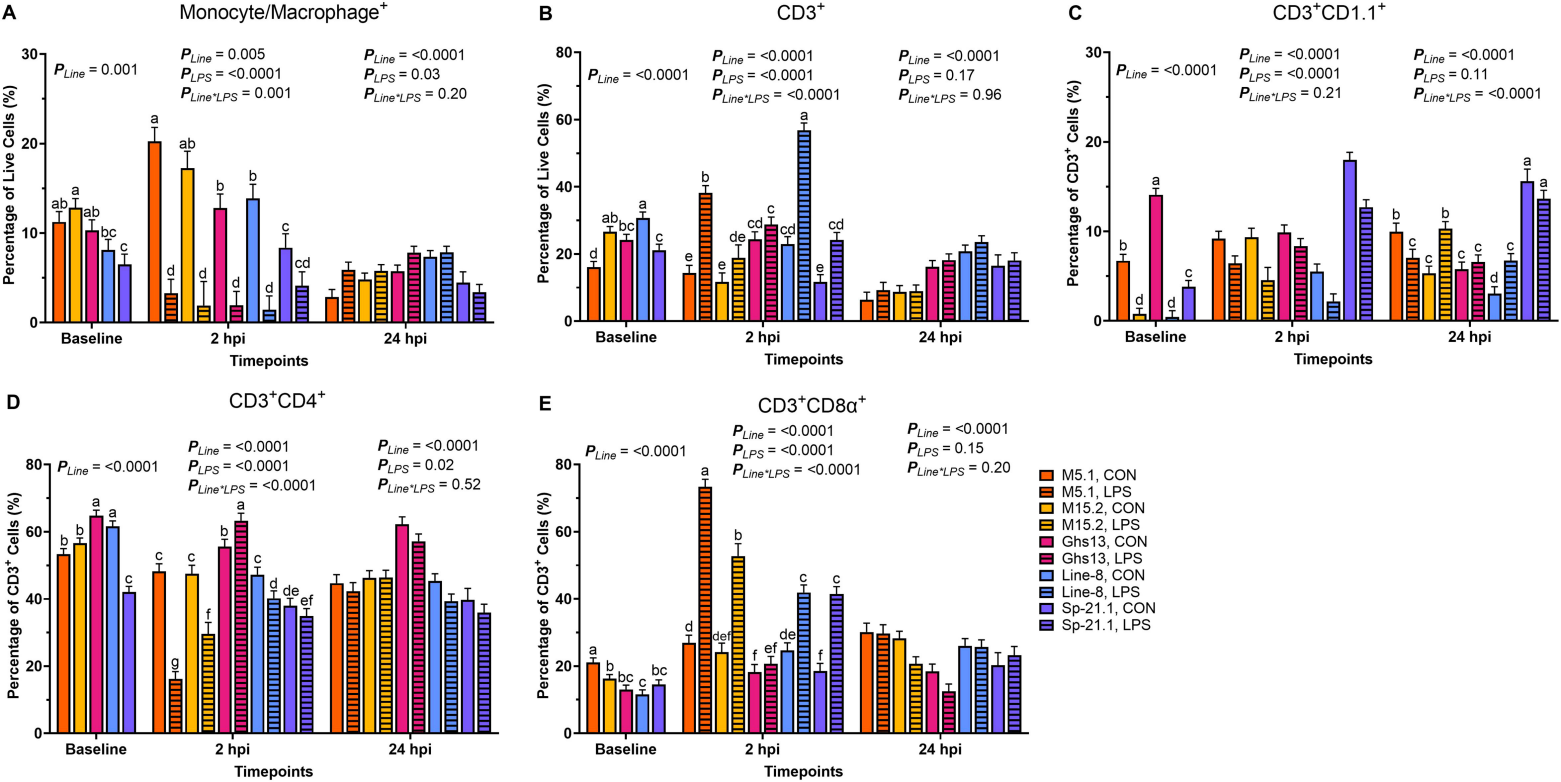
Percentages of **(A)** Monocyte/Macrophage^+^, **(B)** total CD3^+^, **(C)** CD3^+^ CD1.1^+^, **(D)** CD3^+^ CD4^+^, and **(E)** CD3^+^ CD8α^+^ cells isolated from peripheral blood mononuclear cells of 5 highly inbred genetic lines of adult birds (M5.1, M15.2, Ghs13, Line-8, and Sp-21.1) ± 2.4 mg/kg intramuscular LPS injection at baseline, 2 hpi, and 24 hpi. Data represents the mean ± SEM (n = 4 birds/genetic line at baseline; n = 3 birds/treatment for M5.1, Ghs13, Line-8, and Sp-21.1 lines, and n = 2 birds for M15.2 line at 2 hpi; n = 2 birds/treatment for M5.1 and Sp-21.1 lines, and n = 3 birds/treatment for M15.2, Ghs13, and Line-8 lines at 24 hpi). Note that panels A and C are on a smaller scale than the remaining panels. Different letter superscripts denote significant differences within a timepoint *p* ≤ 0.05.

Before inoculation, genetic line significantly influenced all queried cell populations (*p* ≤ 0.001, **Figure 1**). M15.2 birds had 36.6% more monocyte/macrophage^+^ cells than Line-8 birds, while M15.2 and M5.1 displayed similar monocyte/macrophage^+^ levels at baseline (*p* = 0.001, **Figure 1A**). Sp-21.1 birds exhibited 37.1-49.4% fewer monocytes/macrophages than M5.1, M15.2, and Ghs13 at baseline (*p* = 0.001). Line-8 birds had 21.5-47.8% more CD3^+^ cells at baseline than Ghs13, Sp-21.1, and M5.1 (*p* < 0.0001, **Figure 1B**). The M15.2 line also had 20.7-39.7% more CD3^+^ cells than Sp-21.1 and M5.1 at baseline, while Sp-21.1 and M5.1 were not significantly different (*p* < 0.0001). Within baseline CD3^+^ subpopulations, Ghs13 birds had significantly more CD3^+^CD1.1^+^ cells, with a 52.4-96.9% increase compared to all other lines (*p* < 0.0001, **Figure 1C**). M15.2 and Line-8 were similar regarding baseline CD3^+^CD1.1^+^ populations, while M5.1 had 88.4% more CD1.1^+^ cells than M15.2 (*p* < 0.0001). Ghs13 and Line-8 birds had significantly more CD3^+^CD4^+^ cells at baseline, with an 8.1-35.0% increase compared to M15.2, M5.1, and Sp-21.1 (*p* < 0.0001, **Figure 1D**). M15.2 and M5.1 were similar in this regard, and both exhibited 21.1-25.6% more CD3^+^CD4^+^ cells than Sp-21.1 birds (*p* < 0.0001). In baseline CD3^+^CD8α^+^ cell populations, M5.1 birds had the highest levels, with 22.9-45.1% more CD3^+^CD8α^+^ cells than all other lines (*p* < 0.0001, **Figure 1E**). M15.2 also had 28.9% more CD3^+^CD8α^+^ cells than Line-8, while Sp-21.1 and Ghs13 birds had similar levels to Line-8 and M15.2 (*p* < 0.0001).

At 2 hpi, genetic line and injection status interaction was significant for all cell populations except for CD3^+^CD1.1^+^ (*p* ≤ 0.001). Control birds had significantly more monocyte/macrophage^+^ cells in all lines at 2 hpi, except for Sp-21.1 (M5.1 83.7%, M15.2 88.9%, Line-8 89.4%, and Ghs13 84.9%; *p* = 0.001, **Figure 1A**). Total CD3^+^ cells were significantly elevated in LPS-injected Line-8, M5.1, and Sp-21.1 birds compared to their respective controls, with increases of 59.6%, 62.3%, and 51.8%, respectively (*p* < 0.0001, **Figure 1B**). In contrast, Ghs13 and M15.2 birds showed no significant differences in CD3^+^ cells between treatment groups (*p* < 0.0001). Within CD3^+^ subpopulations, control birds had 34.1% more CD3^+^CD1.1^+^ cells than LPS-injected birds at 2 hpi (*p* < 0.0001, **Figure 1C**). Among genetic lines, Sp-21.1 birds exhibited 40.6-74.9% higher CD3^+^CD1.1^+^ cell levels than all other lines at 2 hpi (*p* < 0.0001). M5.1 and M15.2 lines were similar in CD3^+^CD1.1^+^ cells but had 44.5-50.7% more CD3^+^CD1.1^+^ cells than Line-8 birds at 2 hpi (*p* < 0.0001). In CD3^+^CD4^+^ populations, control birds in the M5.1, M15.2, and Line-8 lines had significantly higher CD3^+^CD4^+^ cell levels at 2 hpi compared to their LPS-injected counterparts, being 66.5%, 37.6%, and 14.9% higher, respectively (*p* < 0.0001, **Figure 1D**). Additionally, Ghs13 LPS-injected birds had 12.2% more CD3^+^CD4^+^ cells than their controls at 2 hpi, while Sp-21.1 birds showed no significant difference between treatments (*p* < 0.0001). In contrast, for CD3^+^CD8α^+^ cells, Ghs13 birds were similar across treatments (*p* < 0.0001, **Figure 1E**). However, LPS injection led to significantly increased CD3^+^CD8α^+^ cells in M5.1, M15.2, Line-8, and Sp-21.1 birds compared to their controls, with increases of 63.2%, 54.2%, 41.1%, and 55.2%, respectively (*p* < 0.0001).

At 24 hpi, Ghs13 and Line-8 birds had more monocytes/macrophages+ than M5.1, M15.2, and Sp-21.1 birds (35.5-42.4% M5.1, 21.7-30.1% M15.2, and 41.9-48.2% Sp-21.1, respectively; *p* < 0.0001, **Figure 1A**). Additionally, LPS injection increased monocytes/macrophages^+^ populations by 17.9% at 24 hpi compared to control (*p* = 0.03). Similarly, Line-8 birds had the highest levels of total CD3^+^ cells compared to all other lines at 24 hpi, while Sp-21.1 and Ghs13 birds were similar and fell intermediate (*p* < 0.0001, **Figure 1B**). In CD3^+^ subpopulations at 24 hpi, LPS injection increased CD3^+^CD1.1^+^ cells by 15.2% and 55.0%, respectively, in M15.2 and Line-8 lines compared to control, while M5.1 LPS birds had 29.3% fewer CD3^+^CD1.1^+^ cells than their respective control (*p* < 0.0001, **Figure 1C**). LPS injection also significantly increased CD3^+^CD4^+^ populations by 7.2% compared to control at 24 hpi (*p* = 0.02, **Figure 1D**). Ghs13 had more CD3^+^CD4^+^ cells and fewer CD3^+^CD8α^+^ than all other lines at 24 hpi (22.4-36.7% and 28.7-48.2%, respectively; *p* < 0.0001). M5.1 and M15.2 had similar immune cell profiles across all cell populations at 24 hpi, except for CD3^+^CD8α^+^ levels where M5.1 had 18.3% more CD3^+^CD8α^+^ cells than M15.2 birds (*p* < 0.0001, **Figure 1E**).

### 3.3 Metabolic assays

**Figure 2:**
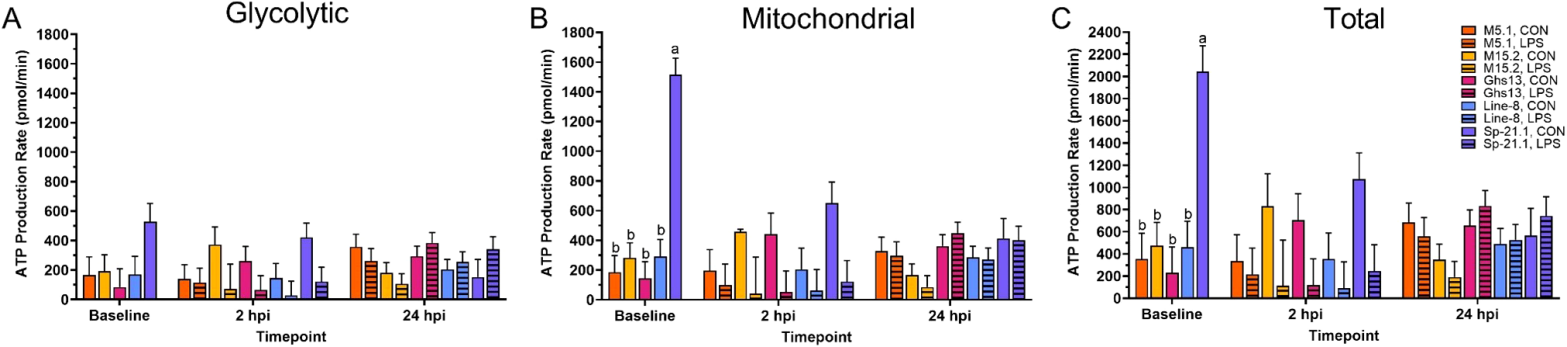
**(A)** Glycolytic, **(B)** mitochondrial, and **(C)** total adenosine triphosphate (ATP) production of peripheral blood mononuclear cells isolated from 5 highly inbred genetic lines of adult birds (M5.1, M15.2, Ghs13, Line-8, and Sp-21.1) ± 2.4 mg/kg intramuscular lipopolysaccharide injection at baseline, 2 hpi, and 24 hpi. Data represent the mean ± SEM (n = 4 birds/genetic line at baseline; n = 3 birds/treatment for M5.1, Ghs13, Line-8, and Sp-21.1 lines, and n = 2 birds for M15.2 line at 2 hpi; n = 2 birds/treatment for M5.1 and Sp-21.1 lines, and n = 3 birds/treatment for M15.2, Ghs13, and Line-8 lines at 24 hpi). Different letters denote significant differences within a timepoint (*p* ≤ 0.05). Note that the y-axis is larger in **(C)**.

No effects in any of the measurements were found with the interaction of line and treatment, therefore, only main effects will be discussed. Glycolytic ATP production did not differ significantly among genetic lines at baseline prior to inoculation (*p* = 0.15, **Table 2**, **Figure 2A**). Genetic line significantly impacted baseline and 24 hpi mitochondrial and total ATP production. At baseline, Sp-21.1 birds produced more mitochondrial and total ATP than all other lines (80.7-90.4% and 76.7-88.7%, respectively; *p* ≤ 0.0002; **Figures 2B, C**). By 24 hpi, M5.1 birds had less mitochondrial and total ATP production compared to Ghs13, M15.2, and Sp-21.1 lines, while Line-8 birds fell intermediate at 24 hpi (59.6-68.9% and 56.7-63.8%, respectively; *p* ≤ 0.04; **Table 2**). LPS treatment had significant impacts only at 2 hpi that were resolved by 24 hpi (**Table 2**). LPS significantly decreased glycolytic, mitochondrial, and total ATP by 70.2%, 80.5%, and 76.3%, respectively, in inoculated birds compared to control at 2 hpi (*p* ≤ 0.01). Likely due to the resolution of LPS inoculation, the effects seen at baseline prior to LPS inoculation resolved by 24 hpi, which suggested the early impacts of LPS on the birds noted at 2 hpi had resolved. In addition, genetic line main effects reemerged at 24 hpi.

**Table 2:** Glycolytic, mitochondrial, and total adenosine triphosphate (ATP) production of peripheral blood mononuclear cells isolated from 5 highly inbred genetic lines of adult birds (M5.1, M15.2, Ghs13, Line-8, and Sp-21.1) administered ± 2.4 mg/kg intramuscular lipopolysaccharide injection at baseline, 2 hpi, and 24 hpi.

ATP Production (pmol/min)	M5.1		M15.2		Ghs13		Line-8		Sp-21.1		Pooled SEM	Adj. *p*-value		
	CON	LPS	CON	LPS	CON	LPS	CON	LPS	CON	LPS		Line^1^	Trt^2^	Line^1^xTrt^2^
Glycolytic														
Baseline	166.3	–	192.8	–	85.2	–	169.5	–	529.0	–	121.1	0.15	–	–
2 hpi	139.4	115.1	372.7	71.0	262.1	64.3	147.1	27.6	421.4	122.4	107.0	0.41	0.01	0.64
24 hpi	358.0	261.6	181.2	105.7	293.7	385.3	204.3	254.3	153.1	342.1	78.9	0.10	0.54	0.46
Mitochondrial														
Baseline	186.2^b^	–	283.1^b^	–	145.2^b^	–	293.0^b^	–	1515.1^a^	–	110.2	<0.0001	–	–
2 hpi	195.7	99.6	458.7	42.8	442.2	53.0	205.4	62.6	651.7	122.6	139.8	0.42	0.01	0.53
24 hpi	327.7	295.6	165.7	86.1	362.3	446.9	284.9	271.4	414.2	401.3	87.6	0.02	0.85	0.87
Total														
Baseline	352.5^b^	–	475.9^b^	–	230.4^b^	–	462.5^b^	–	2044.1^a^	–	227.7	0.0002	–	–
2 hpi	335.2	214.7	831.4	113.8	704.2	117.4	352.5	90.2	1073.2	245.0	260.1	0.42	0.01	0.58
24 hpi	685.7	557.3	346.9	191.8	656.0	832.2	489.2	525.6	567.3	743.4	160.3	0.04	0.84	0.73

Data represent the mean and SEM for all genetic lines at each timepoint (n = 4 birds/genetic line at baseline; n = 3 birds/treatment for M5.1, Ghs13, Line-8, and Sp-21.1 lines, and n = 2 birds for M15.2 line at 2 hpi; n = 2 birds/treatment for M5.1 and Sp-21.1 lines, and n = 3 birds/treatment for M15.2, Ghs13, and Line-8 lines at 24 hpi). Different letter superscripts within a timepoint indicate significance at *p* ≤ 0.05.

^1^Line, Genetic line main effect.

^2^Trt, Challenge main effect.

No interaction effects were found at any timepoint within basal PER, glycolysis, compensatory glycolysis, or post-2-DG acidification rates (**Table 3**). Treatment was the only main effect with significant outcomes. Baseline glycolytic rate assay measures prior to LPS injection were consistent across genetic lines (*p* ≥ 0.20, **Table 3**). At 2 hpi, LPS significantly reduced basal PER, basal glycolysis, compensatory glycolysis, and post-2-dg acidification compared to control birds (68.0%, 70.0%, 70.5%, and 77.7%, respectively; *p* ≤ 0.01). By 24 hpi, no significant differences in glycolytic output were observed due to genetic line, LPS injection, or their interaction (*p* ≥ 0.10). These results mirrored the outcomes found in **Table 2**.

**Table 3:** Basal proton efflux rate (PER), basal glycolysis, compensatory glycolysis, and post-2-DG acidification of peripheral blood mononuclear cells isolated from 5 highly inbred genetic line of adult birds (M5.1, M15.2, Ghs13, Line-8, and Sp-21.1) administered ± 2.4 mg/kg intramuscular lipopolysaccharide injection at baseline, 2 hpi, and 24 hpi.

Measure (pmol/min)	M5.1		M15.2		Ghs13		Line-8		Sp-21.1		Pooled SEM	Adj. *p*-value		
	CON	LPS	CON	LPS	CON	LPS	CON	LPS	CON	LPS		Line^1^	Trt^2^	Line^1^xTrt^2^
Basal PER^3^														
Baseline	165.7	–	151.2	–	101.7	–	144.7	–	210.2	–	30.7	0.23	–	–
2 hpi	137.3	94.6	303.5	85.0	230.3	52.5	143.4	30.7	303.6	95.2	90.9	0.67	0.02	0.84
24 hpi	226.4	219.9	168.9	82.0	220.9	253.6	165.2	194.7	80.3	190.8	53.2	0.16	0.65	0.51
Basal glycolysis														
Baseline	188.2	–	184.0	–	119.5	–	182.2	–	276.2	–	41.9	0.20	–	–
2 hpi	161.9	105.8	355.5	91.2	279.2	58.2	167.1	36.7	372.4	109.3	100.6	0.65	0.02	0.82
24 hpi	257.0	254.9	194.1	92.2	265.4	312.8	206.8	229.3	96.8	235.9	63.5	0.14	0.62	0.51
Compensatory glycolysis														
Baseline	215.3	–	221.1	–	141.3	–	235.6	–	348.7	–	67.9	0.37	–	–
2 hpi	192.7	127.2	414.2	106.4	392.5	68.2	213.5	43.2	467.8	151.3	137.7	0.63	0.02	0.81
24 hpi	319.4	305.0	249.6	100.3	366.9	394.1	314.7	281.0	101.0	290.6	93.4	0.17	0.95	0.59
Post-2-DG														
Baseline	47.2	–	58.7	–	38.3	–	42.7	–	77.0	–	15.7	0.46	–	–
2 hpi	41.7	21.4	83.8	11.1	107.2	21.5	39.8	7.33	100.1	22.7	29.7	0.51	0.01	0.69
24 hpi	52.3	54.1	48.0	12.2	85.0	80.6	54.5	47.0	15.1	51.0	21.1	0.10	0.89	0.65

Data represent the mean and SEM for all genetic lines at each timepoint (n = 4 birds/genetic line at baseline; n = 3 birds/treatment for M5.1, Ghs13, Line-8, and Sp-21.1 lines, and n = 2 birds for M15.2 line at 2 hpi; n = 2 birds/treatment for M5.1 and Sp-21.1 lines, and n = 3 birds/treatment for M15.2, Ghs13, and Line-8 lines at 24 hpi). Different letter superscripts within a timepoint indicate significance at *p* ≤ 0.05.

^1^Line, Genetic line main effect.

^2^Trt, Challenge main effect.

^3^PER, Proton Efflux Rate.

## 4 Discussion

This study evaluated the physiological outcomes, immune profiles, and metabolic phenotypes of PBMC in five highly inbred genetic lines of adult chickens (M5.1, M15.2, Ghs13, Line-8, and Sp-21.1) before and after a single 2.4 mg/kg intramuscular LPS injection.

Genotype-related differences in baseline body weight were expected. Line-8 and Sp-21.1 initial body weights were similar to those reported in previous work by our lab, including adult Line-8 and Sp-21.1 birds (11). Immune cell populations also varied across genetic lines before LPS administration. While M5.1 and M15.2 were similar across monocyte/macrophage and CD3^+^CD4^+^ populations, M5.1 was observed to have higher baseline levels of CD3^+^CD1.1^+^ and CD3^+^CD8α^+^, while M15.2 had elevated total CD3^+^ T cells. However, baseline glycolytic rate and ATP production did not differ between these Major Histocompatibility Complex (MHC) congenic relatives (1, 17). While differences in T cell populations did not contribute to metabolic outcomes between these two lines in this setting, other results suggest that M5.1 may have a more efficient adaptive immune response regarding antigen presentation and cytotoxic activity than M15.2 (18, 19). Notably, M5.1 has been reported to be more resistant to *Eimeria tenella* and *C. perfringens/Eimeria maxima* infections than M15.2, which further supports a more efficient adaptive response in M5.1 than M15.2 (9, 20, 21).

When comparing baseline immune profiles of the Fayoumi and Leghorn Ghs lines, Ghs13 had more CD3^+^, CD3^+^CD1.1^+^, and CD3^+^CD4^+^ cells but fewer CD3^+^CD8α^+^ than M5.1 in the current study. Whereas in younger birds, Fries-Craft et al. noted 21-d-old M5.1 chicks had more peripheral monocytes/macrophages and cytotoxic T cells than the Ghs13 line, while total CD3^+^ and CD3^+^CD4^+^ populations remained similar between the two lines (40.3% and 67.7%, respectively; (16)). Since LPS is a major endotoxin and component of gram-negative bacteria, the lower cytotoxic T cell populations observed in Ghs13 across age may indicate susceptibility to gram-negative bacteria such as *Salmonella*, but further research is needed to confirm this outcome. However, previously research has demonstrated that the Ghs13 line is more susceptible to viral infections, such as Marek’s and Newcastle Disease than M5.1 (10, 22).

Within the Leghorn lines, Line-8 birds exhibited higher baseline CD3^+^ levels than Ghs13, Sp-21.1, and M5.1 but fewer monocytes/macrophages and cytotoxic T cells than M15.2. Similar results were observed here and previously in our lab group, where ~64-wk-old Line-8 birds had 50.9 and 19.5% more total CD3^+^ T cells than Ghs and Sp-21.1 birds at baseline, respectively (**Table 4**, (11)). The Leghorn lines, Ghs13 and Line-8, also had greater helper T-cell activity at baseline than Fayoumi lines and Sp-21.1. Ghs13 birds had the highest proportion of CD3^+^CD1.1^+^ and CD3^+^CD4^+^ cells, suggesting greater potential for antigen presentation and early immune response initiation (18). Meanwhile, Sp-21.1 had fewer monocytes/macrophages at baseline than other lines, which may indicate potential differences in innate immune activation or immune surveillance. Similar results were also observed by Elmore et al., where adult Sp-21.1 had significantly fewer monocytes/macrophages at baseline compared to Ghs and Line-8 birds (11). Within metabolic measures at baseline, glycolytic results suggest that all genetic lines were at similar initial metabolic states before LPS injection. However, Sp-21.1 had significantly higher mitochondrial and total ATP production than all other lines at baseline, indicating a potential preference for mitochondrial production, while all other lines did not indicate a preference for anaerobic or anaerobic pathways. In support of Elmore et al.’s findings, Sp-21.1’s preference for oxidative phosphorylation at baseline could be linked to a more efficient phenotype than all other inbred genetic lines as oxidative phosphorylation produces significantly more ATP per molecule of glucose and produces fewer reactive oxygen species compared to glycolysis (36–38 ATP *vs* 2 ATP per glucose molecule, respectively (11, 23)).

**Table 4:** The relative impact of LPS on cell profiles and metabolic phenotypes of peripheral blood mononuclear cells isolated from 3 highly inbred genetic line of adult birds (Ghs6, Ghs13, Line-8, and Sp-21.1).

LPS dose (mg/kg BW)		Ghs			Line-8			Sp-21.1		
		2.4 (Ghs13)	1 (11) (Ghs6, Ghs13)	2.4/1	2.4	1	2.4/1	2.4	1	2.4/1
Measure	Units	2 hpi	6 hpi	24 hpi^1^	2 hpi	6 hpi	24 hpi^1^	2 hpi	6 hpi	24 hpi^1^
Immune populations	%									
Monocyte/macrophage^+^		↓	↓	-/↑	↓	–	-/-	–	↑	-/-
CD3^+^		–	↓	-/-	↑	↓	-/-	↑	↓	-/-
CD3^+^CD1.1^+^		–	↑	-/-	–	↑	↑/-	–	↑	-/-
CD3^+^CD4^+^		↑	–	-/-	↓	–	-/-	–	–	-/-
CD3^+^CD8α^+^		–	–	-/-	↑	↓	-/↓	↑	↑	-/-
ATP^2^ Production	pmol/min									
Glycolytic		–	–	-/-	–	–	-/-	–	–	-/-
Mitochondrial		–	–	-/-	–	–	-/-	–	↓	-/-
Total		–	–	-/-	–	↑	-/-	–	↓	-/-
Glycolytic Rate	pmol/min									
Basal PER^3^		–	–	-/-	–	–	-/-	–	–	-/-
Basal glycolysis		–	–	-/-	–	–	-/-	–	–	-/-
Compensatory glycolysis		–	–	-/-	–	–	-/-	–	–	-/-
Post-2-DG acidification		–	–	-/-	–	–	-/-	–	–	-/-

^1^Two LPS doses (1 mg/kg and 2 mg/kg BW) are represented at the 24 hpi timepoint, with 2.4 mg/kg BW dose outcomes being displayed on the left side of the column and those from the 1 mg/kg BW dose on the right side.

^2^ATP, adenosine triphosphate production.

^3^PER, Proton Efflux Rate.

Lipopolysaccharide (LPS) was administered intramuscularly at ± 1 mg/kg bodyweight (BW), and outcomes were analyzed at 6 hpi and 24 hpi [data adapted from (11)], or 2.4 mg/kg BW and analyzed at 2 hpi and 24 hpi. Directional arrows indicate significant increases or decreases in the measures (P<0.05), while a dash indicates no significant change (*p* > 0.05).

At 2 hpi, LPS-induced changes in cloacal temperature were not observed but were expected among treatment groups as LPS has been shown to cause severe hyperthermic responses as early as 2 hpi following a 2 mg/kg intramuscular LPS dose (14). Additionally, it has been well reported that 1 mg/kg BW intravenous LPS (*Escherichia coli* O127: B8) or 1 and 2.5 mg/kg BW intraperitoneal LPS (*S*. Typhimurium) injection elicited a fever response as early as 2 hpi in broilers and laying birds (24, 25). Using an identical 1 mg/kg BW intramuscular LPS dosage (*Escherichia coli* O55:B5), our group has demonstrated that LPS significantly lowered cloacal temperatures in ~133-wk-old White Leghorn roosters and trended to reduce cloacal temperature in 4 highly inbred genetic lines (11, 15). While the LPS model was not confirmed by physiological changes induced by LPS injection in the current study, the interaction between genetic line and LPS injection was significant for all immune cell populations except CD3^+^CD1.1^+^ at 2 hpi. Control birds had higher monocyte/macrophage^+^ levels in all genetic lines except Sp-21.1, where LPS-injected birds had similar responses to control. Given the use of an acute LPS challenge and likely previous chronic exposure to environmental LPS, this response is likely a typical sterile inflammatory response, as documented in other sterile inflammation models (26–28). In contrast, our lab previously reported that a single 1 mg/kg intramuscular LPS dose resulted in higher monocyte/macrophage^+^ levels in LPS-stimulated birds than control at 6 hpi (23.8%, (11)). However, in both studies, monocyte/macrophage^+^ levels were significantly decreased in LPS-stimulated Ghs birds at 2 hpi and 6 hpi (**Table 4**, (11)). Therefore, these results may suggest that monocyte/macrophage cells were recruited from circulation upon LPS recognition and were migrating to infected tissues at 2 hpi, while cells had returned to circulation by 6 hpi using a lower LPS dose (i.e., breast and thigh muscle (29)).

In addition, LPS injection significantly increased circulating CD3^+^ T cells in Line-8, M5.1, and Sp-21.1 birds compared to their respective controls, while Ghs13 and M15.2 lines remained similar across treatments at 2 hpi in the present study. In contrast, total CD3^+^ cell levels were significantly lower in LPS-stimulated Line-8 and Sp-21.1 birds 6 h post-1mg/kg intramuscular LPS dose, while Ghs birds remained similar (**Table 4**, (11)). This suggests Line-8 and Sp-21.1 birds had an early response to an increased dose of LPS, while the Ghs line may have delayed responses to immune activation at 2 hpi. Within CD3^+^ subpopulations, control birds had significantly more CD3^+^CD1.1^+^ and CD3^+^CD4^+^ cells than their LPS-stimulated counterparts. Meanwhile, LPS-stimulated birds had higher CD3^+^CD1.1^+^ and CD3^+^CD4^+^ levels 6 h post 1 mg/kg LPS dose (**Table 4**, (11)). However, within Ghs, Line-8, and Sp-21.1, CD3^+^CD1.1^+^ levels remained similar across lines at 2 h post-1 mg/kg LPS dose, while LPS increased CD3^+^CD1.1^+^ cells in all lines at 6 h post-2.4 mg/kg LPS dose. Therefore, 6 hpi is likely to be a more appropriate timepoint to capture shifts CD3^+^CD1.1^+^ cells following a 1-2.4 mg/kg LPS dose in highly inbred genetic lines, but not in adult White Leghorn roosters (15).

In contrast, the LPS dose of 2.4 mg/kg at 2 hpi was a more sensitive model for detecting changes in circulating CD3^+^CD4^+^ and CD3^+^CD8α^+^ for Fayoumi lines and Line-8. For example, LPS stimulation decreased the amount of circulating effect T cells but had the inverse effect on cytotoxic T cells in M5.1, M15.2, and Line-8 lines at 2 hpi in the current study. Meanwhile, no differences were reported in CD3^+^CD4^+^ or CD3^+^CD8α^+^ cells across treatment groups in Ghs and Line-8 at 6 h-post 1 mg/kg LPS dose (**Table 4**, (11)). However, LPS-stimulated Sp-21.1 birds had higher CD3^+^CD8α^+^ levels at both 2 hpi and 6 hpi, indicating an early and strong cytotoxic immune response that is line-specific, despite different LPS doses (30). Additionally, Fayoumi lines were shown to quickly favor cytotoxic T-cell mobilization at 2 hpi at a higher LPS dose, while Ghs birds showed little to no T-cell reactivity to LPS in comparison to all other lines in either model (1 mg/kg LPS at 6 hpi *vs*. 2.4 mg/kg LPS at 2 hpi, **Table 4**). These results indicate that line-specific responses varied following LPS stimulation and immune activation at 2 hpi and 6 hpi. Regarding immunometabolic activity at 2 hpi, LPS significantly decreased ATP production and glycolytic rate across all measures. LPS injection has also been shown to not significantly impact ATP production or glycolytic rate in adult Ghs, Line-8, Sp-21.1, advanced intercross line (AIL-F), or White Leghorn birds 6 h post-1 mg/kg intramuscular LPS dose (11, 15). These results were not expected as immune cells typically switch metabolic pathways from oxidative phosphorylation to glycolysis following LPS stimulation and immune activation to accommodate increased energetic demands (31–34). Therefore, metabolic shifts were expected to favor glycolysis over oxidative phosphorylation during immune activation.

At 24 hpi, distinct differences in immune cell profiles were driven by both LPS- and genetic line-specific responses. Notably, LPS injection significantly increased monocyte/macrophage+ levels from 2 hpi, while control birds maintained significantly higher CD3^+^CD4^+^ levels from 2 hpi compared to LPS-stimulated birds. LPS injection also led to greater CD3^+^CD1.1^+^ levels in M15.2 and Line-8 birds but reduced CD3^+^CD1.1^+^ levels in M5.1 birds at 24 hpi. However, all remaining cell populations were similar across treatment groups in all genetic lines at 24 hpi, suggesting possible LPS clearance or recovery from its earlier effects at 2 hpi. Within genetic lines, Leghorn birds (Ghs13 and Line-8) maintained a more robust monocyte/macrophage response during LPS clearance compared to all other lines. Line-8 birds also continued to have the highest total CD3^+^ populations compared to the remaining lines despite total CD3^+^ populations numerically decreasing from 2 hpi to 24 hpi. Similar findings were reported by Elmore et al., 2025, where Line-8 birds had greater CD3^+^ populations at baseline, 6 hpi and 24 hpi of a 1 mg/kg intramuscular LPS dose than Ghs, Sp-21.1, and AIL-F lines (11). This may indicate that Line-8 birds prefer adaptive immunity to all other lines regardless of LPS injection dose. Interestingly, M5.1 had a stronger cytotoxic immune profile than M15.2 from 2 hpi to 24 hpi. M5.1’s cytotoxic phenotype further supports its known resistance to *Eimeria tenella* and *C. perfringens/Eimeria maxima* infections when compared to M15.2 (9, 20, 21).

Overall, these results demonstrate distinct immune and metabolic phenotypes contributing to varied LPS response among 5 highly inbred genetic lines (M5.1, M15.2, Ghs13, Line-8, and Sp-21.1). These findings support our hypothesis that both immune profiles and PBMC metabolic phenotypes differ by genetic line and influence early response to systemic LPS challenge. The Fayoumi M5.1 displayed a stronger cytotoxic immune profile than M15.2, which aligned with its known resistance to parasitic infections. The Leghorn lines, Ghs13 and Line-8, had greater helper T-cell activity at baseline. Line-8 also had a more robust adaptive immune response 2 h post-LPS injection. Sp-21.1 preferred mitochondrial ATP production at baseline. At 2 h post-2.4 mg/kg LPS dose, immune activation varied by line. Fayoumi birds showed rapid cytotoxic T-cell mobilization, while Ghs13 birds showed a weaker response and possible delayed immune activation. Thus, the 2.4 mg/kg LPS dose at 2 hpi was likely optimal for detecting early immune responses in Fayoumi lines, whereas Ghs birds may be more responsive to a lower 1 mg/kg dose at 6 hpi. Immunometabolic shifts following LPS stimulation did not favor glycolysis as expected, indicating potential line-specific differences in metabolic regulation. By 24 hpi, LPS clearance occurred across most immune cell populations. Future research with increased sample sizes and additional sampling timepoints are warranted to further investigate the relationship between immune profile and immunometabolism following LPS stimulation in these highly inbred genetic lines. These findings highlight the role of genetic background on immune and metabolic responses and provide insight into line-specific immune strategies that may influence resistance to gram-negative bacteria.

## Data Availability

The raw data supporting the conclusions of this article will be made available by the authors, without undue reservation.

## References

[1] Lamont SJ Chen Y Aarts HJ van der Hulst-Van Arkel MC Beuving G Leenstra FR. Endogenous viral genes in thirteen highly inbred chicken lines and in lines selected for immune response traits. Poultry sci. (1992) 71:530–8. doi: 10.3382/ps.0710530 1561219

[2] Aston EJ Wang Y Tracy KE Gallardo RA Lamont SJ Zhou H. Comparison of cellular immune responses to avian influenza virus in two genetically distinct, highly inbred chicken lines. Veterinary Immunol Immunopathol. (2021) 235:110233. doi: 10.1016/j.vetimm.2021.110233 33823380

[3] Deist MS Gallardo RA Bunn DA Kelly TR Dekkers JC Zhou H. Novel mechanisms revealed in the trachea transcriptome of resistant and susceptible chicken lines following infection with Newcastle disease virus. Clin Vaccine Immunol. (2017) 24:e00027–17. doi: 10.1128/CVI.00027-17 PMC5424241 28331077

[4] Jang HJ Monson M Kaiser M Lamont SJ. Induction of chicken host defense peptides within disease-resistant and-susceptible lines. Genes. (2020) 11:1195. doi: 10.3390/genes11101195 33066561 PMC7602260

[5] Wang Y Lupiani B Reddy SM Lamont SJ Zhou H. RNA-seq analysis revealed novel genes and signaling pathway associated with disease resistance to avian influenza virus infection in chickens. Poultry sci. (2014) 93:485–93. doi: 10.3382/ps.2013-03557 24570473

[6] Zhang J Kaiser MG Deist MS Gallardo RA Bunn DA Kelly TR. Transcriptome analysis in spleen reveals differential regulation of response to Newcastle disease virus in two chicken lines. Sci Rep. (2018) 8:1278. doi: 10.1038/s41598-018-19754-8 29352240 PMC5775430

[7] Van Goor A Slawinska A Schmidt CJ Lamont SJ. Distinct functional responses to stressors of bone marrow derived dendritic cells from diverse inbred chicken lines. Dev Comp Immunol. (2016) 63:96–110. doi: 10.1016/j.dci.2016.05.016 27238770

[8] Kim DK Lillehoj HS Hong YH Park DW Lamont SJ Han JY. Immune-related gene expression in two B-complex disparate genetically inbred Fayoumi chicken lines following Eimeria maxima infection. Poultry Sci. (2008) 87:433–43. doi: 10.3382/ps.2007-00383 18281568

[9] Lee SH Dong X Lillehoj HS Lamont SJ Suo X Kim DK. Comparing the immune responses of two genetically B-complex disparate Fayoumi chicken lines to Eimeria tenella. Br Poultry Sci. (2016) 57:165–71. doi: 10.1080/00071668.2016.1141172 26942865

[10] Schilling MA Memari S Cavanaugh M Katani R Deist MS Radzio-Basu J. Conserved, breed-dependent, and subline-dependent innate immune responses of Fayoumi and Leghorn chicken embryos to Newcastle disease virus infection. Sci Rep. (2019) 9:7209. doi: 10.1038/s41598-019-43483-1 31076577 PMC6510893

[11] Elmore KM Lamont SJ Bobeck EA. Immune cell profile and metabolic preferences following intramuscular lipopolysaccharide injection of highly inbred and advanced intercross genetic lines. Front Veterinary Sci. (2025) 12:1592021. doi: 10.3389/fvets.2025.1592021 PMC12170644 40530037

[12] Bertani B Ruiz N. Function and biogenesis of lipopolysaccharides. Ecosal plus. (2018) 8:10–128. doi: 10.1128/ecosalplus.esp-0001-2018 PMC6091223 30066669

[13] Lieboldt MA Frahm J Halle I Schrader L Weigend S Preisinger R. Haematological and febrile response to Escherichia coli lipopolysaccharide in 12-week-old cockerels of genetically diverse layer lines fed diets with increasing L-arginine levels. J Anim Physiol Anim Nutr. (2017) 101:743–54. doi: 10.1111/jpn.12466 27080348

[14] Lieboldt MA Frahm J Halle I Görs S Schrader L Weigend S. Metabolic and clinical response to Escherichia coli lipopolysaccharide in layer pullets of different genetic backgrounds supplied with graded dietary L-arginine. Poult Sci. (2016) 95:595–611. doi: 10.3382/ps/pev359 26740139

[15] Elmore KM Bobeck EA. Metabolic and immune profiles of 1-year and 2.5+ year-old white leghorn roosters following intramuscular lipopolysaccharide injection. Front Veterinary Sci. (2025) 12:1547807. doi: 10.3389/fvets.2025.1547807 PMC11880223 40046416

[16] Fries-Craft K Lamont SJ Bobeck EA. Implementing real-time immunometabolic assays and immune cell profiling to evaluate systemic immune response variations to Eimeria challenge in three novel layer genetic lines. Front Veterinary Sci. (2023) 10:1179198. doi: 10.3389/fvets.2023.1179198 PMC10153671 37143494

[17] Kaiser M Kaufman J Lamont SJ. Different MHC class I cell surface expression levels in diverse chicken lines, associations with B blood group, and proposed relationship to antigen-binding repertoire. Poultry Sci. (2025) 104:104569. doi: 10.1016/j.psj.2024.104569 39642749 PMC11665679

[18] Porcelli SA Modlin RL. The CD1 system: antigen-presenting molecules for T cell recognition of lipids and glycolipids. Annu Rev Immunol. (1999) 17:297–329. doi: 10.1146/annurev.immunol.17.1.297 10358761

[19] Erf GF. Cell-mediated immunity in poultry. Poultry sci. (2004) 83:580–90. doi: 10.1093/ps/83.4.580 15109055

[20] Kim DK Lillehoj HS Jang SI Lee SH Hong YH Lamont SJ. Genetically disparate Fayoumi chicken lines show different response to avian necrotic enteritis. J Poultry Sci. (2015) 52:245–52. doi: 10.2141/jpsa.0140203

[21] Truong AD Hong Y Ban J Park B Hoang TC Hong YH. Analysis of differentially expressed genes in necrotic enteritis-infected fayoumi chickens using RNA sequencing. J Poultry Sci. (2017) 54:121–33. doi: 10.2141/jpsa.0160053 PMC7477130 32908417

[22] Lakshmanan N Kaiser MG Lamont SJ. Marek’s disease (MD) resistance in MHC-congenic lines from Leghorn and Fayoumi breeds. In: Silva RF Cheng HH Coussens PM Lee LF Velicer LF, editors. Current Research on Marek’s Disease. American Association of Avian Pathologists, Kennett Square (PA (1997). p. 57–62.

[23] Yetkin-Arik B Vogels IM Nowak-Sliwinska P Weiss A Houtkooper RH Van Noorden CJ. The role of glycolysis and mitochondrial respiration in the formation and functioning of endothelial tip cells during angiogenesis. Sci Rep. (2019) 9:12608. doi: 10.1038/s41598-019-48676-2 31471554 PMC6717205

[24] Jones CA Edens FW Denbow DM. Rectal temperature and blood chemical responses of young chickens given E. coli endotoxin Poult Sci. (1981) 60:2189–94. doi: 10.3382/ps.0602189 7036123

[25] Leshchinsky TV Klasing KC. Divergence of the inflammatory response in two types of chickens. Dev Comp Immunol. (2001) 25:629–38. doi: 10.1016/S0145-305X(01)00023-4 11472784

[26] De Boever S Croubels S Meyer E Sys S Beyaert R Ducatelle R. Characterization of an intravenous lipopolysaccharide inflammation model in broiler chickens. Avian Pathol. (2009) 38:403–11. doi: 10.1080/03079450903190871 19937527

[27] Rock KL Latz E Ontiveros F Kono H. The sterile inflammatory response. Annu Rev Immunol. (2009) 28:321–42. doi: 10.1146/annurev-immunol-030409-101311 PMC4315152 20307211

[28] Rubartelli A Lotze MT Latz E Manfredi A. Mechanisms of sterile inflammation. Front Immunol. (2013) 4:398. doi: 10.3389/fimmu.2013.00398 24319446 PMC3837241

[29] Imhof BA Aurrand-Lions M. Adhesion mechanisms regulating the migration of monocytes. Nat Rev Immunol. (2004) 4:432–44. doi: 10.1038/nri1375 15173832

[30] Miller RA. Aging and immune function. Int Rev Cytol. (1991) 124:187–215. doi: 10.1016/S0074-7696(08)61527-2 2001916

[31] Warburg O Gawehn K Geissler AW. Metabolism of leukocytes. Zeitschrift fur Naturforschung Teil B, Chemie, Biochemie, Biophysik, Biologie und verwandte Gebiete. (1958) 13:515–6. 13593654

[32] Palsson-McDermott EM O’neill LA. The Warburg effect then and now: from cancer to inflammatory diseases. BioEssays. (2013) 35:965–73. doi: 10.1002/bies.201300084 24115022

[33] Martinez FO Helming L Gordon S. Alternative activation of macrophages: an immunologic functional perspective. Annu Rev Immunol. (2009) 27:451–83. doi: 10.1146/annurev.immunol.021908.132532 19105661

[34] Rambold A Pearce E. Mitochondrial dynamics at the interface of immune cell metabolism and function. Trends Immunol. (2018) 39:6–18. doi: 10.1016/j.it.2017.08.006 28923365

